# Grape Extracts Inhibit Multiple Events in the Cell Biology of Cholera Intoxication

**DOI:** 10.1371/journal.pone.0073390

**Published:** 2013-09-05

**Authors:** Srikar Reddy, Michael Taylor, Mojun Zhao, Patrick Cherubin, Sandra Geden, Supriyo Ray, David Francis, Ken Teter

**Affiliations:** 1 Burnett School of Biomedical Sciences, College of Medicine, University of Central Florida, Orlando, Florida, United States of America; 2 Lake Highland Preparatory School, Orlando, Florida, United States of America; 3 The Center for Infectious Disease Research and Vaccinology, Department of Veterinary and Biomedical Sciences, South Dakota State University, Brookings, South Dakota, United States of America; Columbia University, United States of America

## Abstract

*Vibrio cholerae* produces cholera toxin (CT), an AB_5_ protein toxin that is primarily responsible for the profuse watery diarrhea of cholera. CT is secreted into the extracellular milieu, but the toxin attacks its Gsα target within the cytosol of a host cell. Thus, CT must cross a cellular membrane barrier in order to function. This event only occurs after the toxin travels by retrograde vesicular transport from the cell surface to the endoplasmic reticulum (ER). The catalytic A1 polypeptide then dissociates from the rest of the toxin and assumes an unfolded conformation that facilitates its transfer to the cytosol by a process involving the quality control system of ER-associated degradation. Productive intoxication is blocked by alterations to the vesicular transport of CT and/or the ER-to-cytosol translocation of CTA1. Various plant compounds have been reported to inhibit the cytopathic activity of CT, so in this work we evaluated the potential anti-CT properties of grape extract. Two grape extracts currently sold as nutritional supplements inhibited CT and *Escherichia coli* heat-labile toxin activity against cultured cells and intestinal loops. CT intoxication was blocked even when the extracts were added an hour after the initial toxin exposure. A specific subset of host-toxin interactions involving both the catalytic CTA1 subunit and the cell-binding CTB pentamer were affected. The extracts blocked toxin binding to the cell surface, prevented unfolding of the isolated CTA1 subunit, inhibited CTA1 translocation to the cytosol, and disrupted the catalytic activity of CTA1. Grape extract could thus potentially serve as a novel therapeutic to prevent or possibly treat cholera.

## Introduction

Cholera toxin (CT), produced by *Vibrio cholerae*, is an AB_5_ toxin responsible for the profuse, life-threatening diarrhea of cholera [1]–[3]. This physiological response results from ADP-ribosylation of Gsα by the catalytic A1 subunit of CT (CTA1). To reach its Gsα target, CT travels from the cell surface to the endoplasmic reticulum (ER) as an intact AB_5_ holotoxin [4]. CT initially interacts with the target cell through binding of its B homopentamer to GM1 gangliosides in the host plasma membrane. Surface-bound CT is then internalized into the host cell through an endocytic mechanism involving lipid rafts [5], [6]. The majority of endocytosed toxin is degraded in the lysosomes, but the functional pool of toxin is instead delivered to the *trans*-Golgi network en route to the ER [7]–[10]. Reduction of the CTA1/CTA2 disulfide bond occurs in the ER [11], [12], which is then followed by the separation of CTA1 from CTA2/CTB_5_ by protein disulfide isomerase (PDI) [13], [14]. Dissociation of the CTA1 subunit leads to its spontaneous unfolding and subsequent recognition by the quality control system of ER-associated degradation (ERAD) [15]–[18]. Misfolded or misassembled proteins are recognized by ERAD, exported from the ER to the cytosol, and degraded by the cytosolic ubiquitin-proteasome system [19]. CTA1 exploits ERAD for its ER-to-cytosol translocation [20], [21], but the lack of lysine residues in the CTA1 polypeptide allows the translocated toxin to avoid ubiquitination and subsequent degradation by the 26S proteasome [17], [22], [23]. Instead, cytosolic CTA1 regains an active conformation and attacks its Gsα target.

Disruptions to the CT trafficking/translocation itinerary will block CTA1 passage into the cytosol and thereby generate a toxin-resistant phenotype. Neutralizing antibodies will prevent CT binding to the cell surface and, thus, productive intoxication [24]. Dissolution of lipid rafts with filipin or methyl β-cyclodextrin prevents toxin endocytosis and/or transport to the Golgi apparatus [5], [6], [25]. Endosome-to-Golgi and Golgi-to-ER transport steps are inhibited by the expression of dominant negative ARF1 or Sar1 proteins [26], [27]. Brefeldin A (BfA) blocks toxin transport to the ER [8]–[10], [26]. The loss of ERdj3 [28] or BiP [29] function will prevent CTA1 translocation to the cytosol, as will ERAD dysfunction [20]. An inhibition of CTA1 unfolding resulting from treatment with 4-phenylbutyric acid, 10% glycerol, or mildly acidic pH will also prevent the ER-to-cytosol export of CTA1 [15], [16], [18]. Although these studies have helped map the intracellular route of CT intoxication, they have only identified a few valid options for therapeutic development. Ongoing efforts continue to search for therapeutic agents that could be effective against cholera and, preferably, other toxin-mediated diseases as well.

We recently screened a select panel of plant compounds for inhibitory activity against Shiga toxin (ST), another AB-type ER-translocating toxin [30]. Plant compounds were screened because polyphenols and other plant compounds have been shown to inhibit CT intoxication and the release of ST from *Escherichia coli* O157:H7 [31]–[34]. Grape seed extract and grape pomace (i.e., skin) extract each conferred substantial cellular resistance to ST when applied simultaneously with the toxin to cultured Vero cells [30]. Both extracts are Generally Recognized as Safe by the United States Food and Drug Administration and are sold as nutritional supplements under the names MegaNatural Gold (grape seed extract) and MegaNatural GSKE (grape pomace extract). In this work, we report the extracts inhibited CT activity against cultured cells and intestinal loops. Application of the extracts up to an hour after toxin exposure still generated a toxin-resistant phenotype in cultured cells. Toxin resistance resulted from extract-induced disruptions to multiple steps of the intoxication process, including CTB binding to the cell surface, CTA1 unfolding in the ER, CTA1 translocation to the cytosol, and CTA1 ADP-ribosylation activity. Toxin trafficking to the ER, CTA1/CTA2 redox status, and CTA1 separation from the holotoxin were not affected by the extracts. These observations indicate the grape extracts block specific events in the cell biology of CT intoxication and suggest a new anti-toxin therapeutic use for two existing nutritional supplements.

## Materials and Methods

### Ethics Statement

Intestinal loop experiments were performed with approval from the South Dakota State University Institutional Animal Care and Use committee, protocol number 11-008A. Animals were tranquilized and anesthetized with 6 mg/kg of Telazol and maintained on isoflurane gas anesthesia, with oxygen by mask from an anesthetic machine for the entire experimental period. The experiment was terminated with euthanasia done in accordance with the recommendations of the American Veterinary Medical Association.

### Statistics

As indicated, data are presented as averages ± standard deviations or means ± standard errors of the means. Data were analyzed by one-way ANOVA using StatPlus from AnalystSoft, Inc. (Vancouver, BC). A *p* value of <0.05 was considered statistically significant.

### Materials

Digitonin was purchased from Calbiochem (La Jolla, CA). CT and the *E. coli* heat-labile toxin (LT) were purchased from List Biologicals (Campbell, CA). The anti-KDEL antibody was purchased from Stressgen (San Diego, CA). The CTA1/CTA2 heterodimer, CTB pentamer, fluorescein isothiocyanate-conjugated CTB pentamer (FITC-CTB), GM1, BfA, thermolysin, α-casein, PDI, and anti-CTA1 antibody were purchased from Sigma-Aldrich (St. Louis, MO). Cholesterol and phospholipids were purchased from Avanti Polar Lipids (Alabaster, AL). Purified phenolic compounds were purchased from ChromaDex, Inc. (Irvine, CA). Grape seed and grape pomace extracts, provided by Polyphenolics, Inc. (Madera, CA), were used at 100 µg/mL concentrations for all experiments. Previous work has demonstrated the extracts are non-toxic to cultured cells at concentrations up to 500 µg/mL [30].

### Cell Culture Toxicity Assays

CHO-K1 cells (ATCC #CCL-61) grown to 80% confluency in 24-well plates were used for toxicity assays. Toxin-treated cells were solubilized in 0.25 mL ice-cold HCl:EtOH (1∶100) for 15 min at 4°C. Cell extracts were transferred to microcentrifuge tubes and allowed to air dry. The dried extracts were reconstituted in assay buffer, and cAMP levels were quantified using a commercial kit (GE Healthcare, Piscataway, NJ). The basal level of cAMP from unintoxicated cells was background subtracted from the experimental values before presenting the data as percentages of the maximal cAMP response for the experiment.

### Intestinal Loop Assay

One week old pigs were anesthetized, and 3–4 loops per condition were prepared. Each ligated segment was approximately 6 cm in length, with intervening 3 cm loops between the experimental loops. A 1 mL volume of phosphate-buffered saline (PBS) lacking or containing the stated extracts and/or toxins was injected into the loops. At 8 h post-injection, the pigs were euthanized, and each excised loop was measured for length and fluid accumulation. The ratio of fluid accumulation to segment length was calculated as a measure of toxin activity.

### Assay for CTB Binding to the Cell Surface

CHO cells grown to 75% confluency in 96-well clear-bottom black-walled plates (Greiner Bio-One, Monroe, North Carolina) were incubated with 1 µg/mL of FITC-CTB at 4°C under the stated conditions. FITC-CTB was removed from the medium, and the cells were washed with PBS before measurement with a Synergy 2 plate reader (BioTek, Winooski, VT) using 485/20 nm excitation and 528/20 nm emission wavelength filter cubes. The fluorescent signal from CHO cells that had not been exposed to FITC-CTB was background subtracted from experimental values before presenting the data as percentages of the maximum fluorescent signal for the experiment.

### Use of Large Unilamellar Vesicles (LUVs) for SPR Studies

LUVs prepared as previously described [35] were formulated to mimic the composition of the lipid raft binding site for CT: 5% GM1, 30% cholesterol, 40% 1-Palmitoyl-2-Linoleoyl-*sn*-Glycero-3-Phosphoethanolamine, 15% 1-Palmitoyl-2-Linoleoyl-*sn*-Glycero-3-Phospho-L-Serine, and 10% 1-Palmitoyl-2-Linoeoyl-*sn*-Glycero-3-Phosphocholine. Control LUVs lacking GM1 increased the amount of 1-Palmitoyl-2-Linoleoyl-*sn*-Glycero-3-Phosphoethanolamine to 45%. To append the LUVs on an SPR sensor, a Reichert self-assembled monolayer slide was activated with EDC/NHS as previously described [36]. 25 mg of phytosphingosine dissolved in 2.5 mL dimethyl sulfoxide was mixed with 2.5 mL of 40 mM sodium acetate (pH 5.2) and perfused over the activated slide for 45 min at a flow rate of 41 µL/min. Unbound tethers left on the slide were then inactivated with a 5 min perfusion of 1 M ethanolamine (pH 8.5). A 1.6 mM concentration of LUVs was then perfused over the slide for 5 min at a flow rate of 41 µL/min. A subsequent 10 min perfusion with PBS containing 0.05% Tween 20 was used to establish a stable baseline before perfusion of CT over the LUV-coated sensor.

### Assay for Holotoxin Disassembly

CT was appended to a GM1-coated SPR sensor slide as previously described [18]. PDI (0.1 mM) was then perfused over the CT-coated sensor in PBS containing 0.05% Tween 20, 1 mM GSH, and either grape seed or grape pomace extract. After stabilization of the SPR signal, PDI was removed from the perfusion buffer and replaced with sequential additions of anti-CTA1 (1∶5,000 dilution) and anti-KDEL (1∶10,000 dilution) antibodies.

### Assay for Toxin Transport to the ER

As previously described [18], HeLa cells (ATCC #CCL-2) grown to 80% confluency in 6-well plates were treated with 100 ng/mL of GM1 for 1 h at 37°C. The cells were washed and exposed to 1 µg/mL CT for 30 min at 4°C. This temperature allows toxin binding to the plasma membrane but blocks endocytosis of the surface-bound toxin. The cells were then washed and incubated in toxin-free medium at 37°C, a temperature that permits toxin endocytosis and intracellular transport. Extracts were only added 15, 30, or 60 minutes after warming to 37°C. With this protocol, the extracts would not block toxin binding to the plasma membrane because they were only added to the medium after endocytosis of the surface-bound toxin had already occurred. After a total of 2 h at 37°C, cells were lifted from the plates with 0.5 mM ethylenediaminetetraacetic acid in PBS pH 7.4. Cell pellets obtained after low speed centrifugation were resuspended in 0.1 mL HCN buffer (50 mM Hepes pH 7.5, 150 mM NaCl, 2 mM CaCl_2_, 10 mM N-ethylmaleimide, and a protease inhibitor cocktail) containing 0.04% digitonin. After 10 min at 4°C, the semi-permeabilized cell extract was partitioned into membrane and cytosolic fractions by centrifugation. The membrane pellet was resuspended in 120 µL of 1×sample buffer, and a 25 µL sample was resolved by non-reducing sodium dodecyl sulfate polyacrylamide gel electrophoresis (SDS-PAGE) with a 15% polyacrylamide gel. Western blot analysis with a primary rabbit anti-CTA1 antibody at 1∶20,000 dilution and a secondary horseradish peroxidase-conjugated goat anti-rabbit IgG antibody at 1∶20,000 dilution was used to detect the reduced and disulfide-linked forms of CTA1. HeLa cells were used for this assay rather than CHO cells because, in our experience, the quantity of CTA1 which reaches the ER of CHO cells is often below the threshold of Western blot detection. An overnight toxicity assay confirmed that either grape extract could inhibit CT activity against HeLa cells (data not shown).

### CTA1 Translocation and Secretion Assays

HeLa cells exposed to GM1 and CT were processed as described above for the toxin transport assay, except (i) media samples were collected before lifting the cells from the plates; (ii) the cell pellet was resuspended in 1 mL HCN/digitonin buffer; and (iii) the cytosolic fraction was collected and diluted to a final volume of 1 mL in HCN buffer. Media samples, cytosolic fractions, and CTA standards were perfused over an SPR sensor coated with an anti-CTA1 antibody as previously described [18]. As with the assay for toxin transport to the ER, this protocol has been optimized for HeLa cells rather than the CHO cells which produce weaker signals for both ER-localized and cytosolic CTA1 [16], [18], [36].

### Protease Sensitivity Assay

A master mix of the purified CTA1/CTA2 heterodimer was placed in 20 mM sodium phosphate buffer pH 7.4 containing 10 mM β-mercaptoethanol. Parallel master mixes were prepared in the presence of either grape seed or grape pomace extract. Control experiments using non-reducing SDS-PAGE established that the grape extracts did not reduce the CTA1/CTA2 disulfide bond, nor did they prevent disulfide reduction by β-mercaptoethanol. Aliquots from each master mix, corresponding to 1 µg toxin samples, were incubated at 4°C, 25°C, 33°C, 37°C, or 41°C for 1 h and were then placed on ice. Thermolysin (prepared as a 10×stock in 50 mM CaCl_2_ and 100 mM HEPES, pH 8.0) was then added to a final concentration of 0.04 mg/mL for a 1 h incubation at 4°C. The samples were resolved by SDS-PAGE (15% polyacrylamide gels) and visualized with Coomassie staining. Since all protease treatments were conducted at 4°C, differential degradation of the toxin samples could not be attributed to temperature-dependent protease activity. Instead, differences were due to the temperature-induced alterations of CTA1 structure which occurred prior to the 4°C protease treatment [17].

### ADP-ribosylation Assay

Diethylamino(benzylidine-amino)guanidine (DEA-BAG), a substrate for the ADP-ribosyltransferase activity of CTA1 [37], was synthesized by heating 500 mL of 0.11 M aminoguanidine bicarbonate (Sigma-Aldrich) pH 6.5 to 60°C after filtration removed insoluble solids. 4-Diethylaminobenzaldehyde (Acros Organics) was added in 200 proof ethanol to make a 1∶1 molar ratio with aminoguanidine bicarbonate, and the solution was stirred at room temperature for 16 h. The DEA-BAG precipitate was harvested by filtration. The remaining solution was then evaporated and cooled to promote further precipitate formation. The collected DEA-BAG precipitate was lyophilized and stored at −80°C.

Dilutions of CTA1/CTA2 were placed in 200 mM Na/K phosphate buffer pH 7.5 containing 20 mM DTT, 0.1 mg/mL bovine serum albumin, 10 mM NAD, and 2 mM DEA-BAG. Bio-Rad (Hercules, CA) AG-50W-X4 ion exchange resin was added to the reaction mixture after 2 h at 25°C. DEA-BAG loses its ability to bind this resin after ADP-ribosylation, so unmodified DEA-BAG was removed from the reaction mixture by pelleting the resin in a microcentrifuge tube. The relative amount of ADP-ribosylated DEA-BAG remaining in the supernatant was determined by measuring the intrinsic fluorescence of DEA-BAG with a Synergy 2 plate reader at 360 nm excitation and 460 emission wavelengths.

## Results

### Grape Extracts Block Intoxication of Cultured Cells and Intestinal Loops

**Figure 1:**
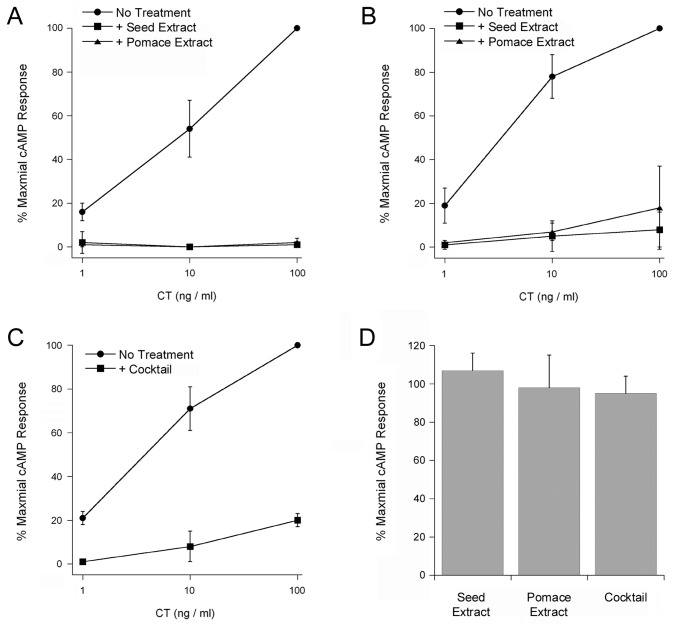
Grape extracts inhibit cholera intoxication of culture cells. (A–B) CHO cells were incubated for 2 h (A) or 18 h (B) with various concentrations of CT in the absence or presence of grape extract before cAMP levels were quantified. (C) CHO cells were incubated for 2 h with various concentrations of CT in the absence or presence of a chemically defined phenolic cocktail before cAMP levels were quantified. (D) CHO cells were incubated for 2 h with 100 µM forskolin in the absence or presence of grape seed extract, grape pomace extract, or phenolic cocktail before cAMP levels were quantified. For all panels, data are presented as percentages of the maximal cAMP response for the experiment (i.e., the cAMP level obtained from untreated cells exposed to 100 ng/mL of CT or to 100 µM forskolin). The averages ± standard deviations of 3–4 independent experiments with triplicate samples are shown. In panels A–C, all experimental conditions were significantly different from the No Treatment control at every matched toxin concentration (1-way ANOVA, *p*<0.05).

Our previous work identified grape seed and grape pomace extracts as potent inhibitors of ST [30]. To determine whether the extracts could inhibit other toxins as well, we exposed CHO cells to various concentrations of CT in the absence or presence of grape extract. Cells exposed to either grape seed or grape pomace extract exhibited high levels of resistance to CT after both short-term (Fig. 1A) and long-term (Fig. 1B) toxin exposures. Extract-treated cells were also resistant to *E. coli* LT, another AB_5_-type toxin that is highly related to CT [1], [2]: an overnight incubation with 100 ng/mL of LT and either grape extract generated no more than 8% of the cAMP response obtained from cells incubated with LT alone (*n* = 2). Grape extracts are highly enriched in polyphenolic compounds with established anti-toxin properties [38]–[42]. We therefore hypothesized that the phenolic constituents of grape extract represent a source of anti-toxin compounds. To test this hypothesis, we applied a defined cocktail of purified phenolic compounds (Table 1) to toxin-treated CHO cells (Fig. 1C). This cocktail conferred substantial resistance to CT, which suggested a phenolic compound(s) was at least partially responsible for the extract-induced inhibition of CT activity against cultured cells. Neither of the grape extracts nor the phenolic cocktail inhibited cAMP production in cells treated with forskolin, an agonist of adenylate cyclase (Fig. 1D).

**Table 1:** Composition of the chemically defined phenolic cocktail.

Compound
Caftaric acid
Catechin
Catechin gallate
Cyanidin
Delphinidin
Epicatechin
Epicatechin gallate
Epigallocatechin gallate
Gallic Acid
Kaempferol
Kuromanin
Malvin chloride
Oenin chloride
Peonidin
Petunidin
Protocatechin
Quercitrin
Resveratrol

Eighteen phenolic compounds commonly found in grape extract [37]–[40] were mixed in Ham’s F-12 medium. The toxicity assay of Figure 1C used a final working concentration of 5 µg/mL for each listed compound.

**Figure 2:**
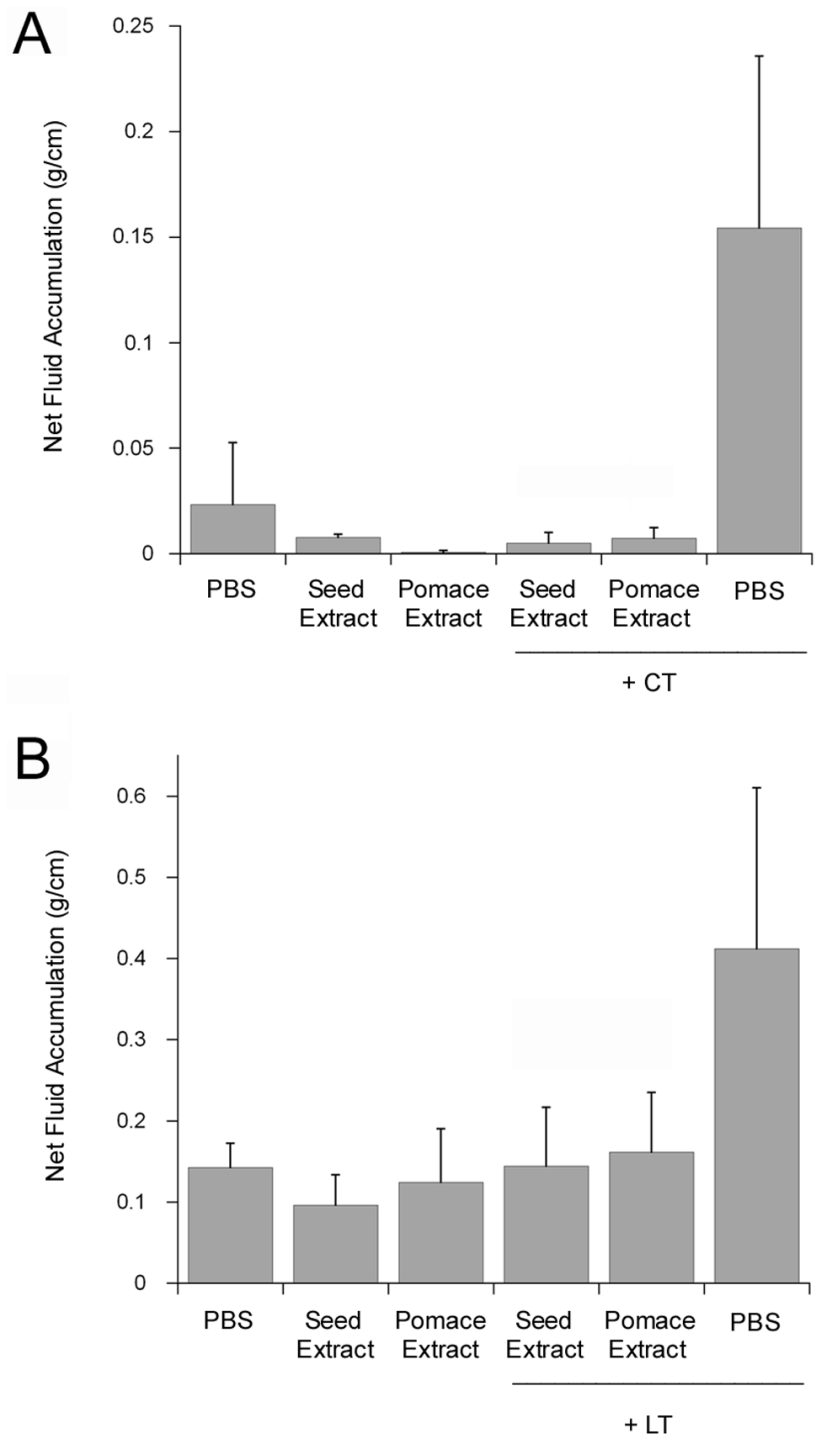
Grape extracts inhibit the diarrheatic response to CT and LT. A ligated intestinal loop assay in neonatal pigs was used to monitor net fluid accumulation in response to an 8 h challenge with CT (A) or LT (B). Loops were injected with PBS alone or PBS containing 10 mg grape seed extract, 1 mg grape pomace extract, and/or 10 µg toxin as indicated. Data represent the averages ± standard deviations of 3–4 replicate loops in a single pig for each toxin/experiment. Statistically significant differences between loops challenged with toxin in the absence vs. presence of either grape extract were detected for both CT (*p*<0.001) and LT (*p*<0.01) by ANOVA. No significant differences were noted between (i) loops treated with PBS alone and any extract-treated loop; (ii) loops treated with seed extract and loops treated with both seed extract and toxin; or (iii) loops treated with pomace extract and loops treated with both pomace extract and toxin.

The grape extracts acted as inhibitors in an intestinal model of CT and LT intoxication as well (Fig. 2). For both CT (Fig. 2A) and LT (Fig. 2B), the toxin-induced fluid accumulation was completely negated by co-injection with either grape extract. Statistically significant differences between toxin-treated and toxin/extract-treated loops were detected for both CT and LT. Thus, the extracts could effectively block CT/LT intoxication of both cultured cells and intestinal loops. Additional experiments were performed in order to identify the molecular basis for extract-induced resistance to CT.

### Grape Extracts Block Toxin Interactions with GM1

**Figure 3:**
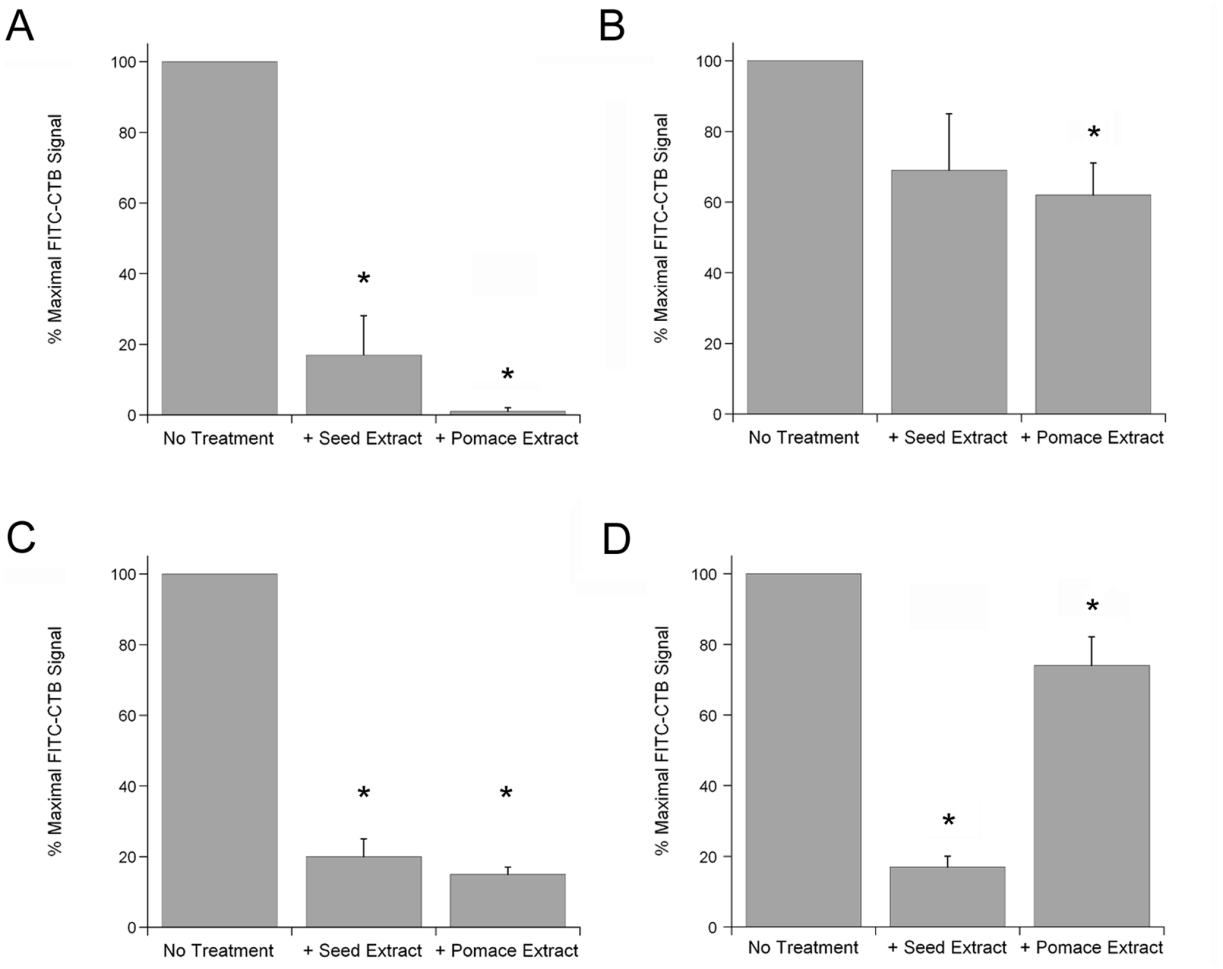
Grape extracts inhibit toxin binding to the cell surface. CHO cells were placed on ice and exposed to FITC-CTB for 30–60 min. The cells were washed with PBS to remove unbound toxin, and fluorescent output was determined with a plate reader. (A) Cells were co-incubated with FITC-CTB and grape seed or pomace extract for 60 min. (B) Cells were exposed to grape extract for 30 min. The extracts were removed by washing with PBS, and the cells were then exposed to FITC-CTB for 30 min. (C) Cells were exposed to FITC-CTB for 30 min. Unbound toxin was removed by washing with PBS, and the cells were then exposed to grape extract for 30 min. (D) A mixture of FITC-CTB and grape extract was placed in a 3500 MWCO dialysis cup. After an overnight dialysis, FITC-CTB was applied to cells for 60 min. All steps of all experiments were performed at 4°C, and all experiments contained a No Treatment control in which FITC-CTB was not exposed to grape extract but was otherwise treated identically to the extract-treated samples. For all panels, data are presented as percentages of the maximal FITC-CTB signal obtained from the No Treatment control. The means ± standard errors of the means of 4 independent experiments with 6 replicates per condition are shown. Asterisks denote statistically significant differences between untreated and extract-treated cells (1-way ANOVA, *p*<0.01).

Plant compounds can affect intoxication through an inhibition of toxin binding to the plasma membrane [31], [43], [44]. To determine if the grape extracts blocked CT binding at the cell surface, we incubated CHO cells at 4°C with a FITC-conjugated CTB pentamer in the absence or presence of grape extract (Fig. 3). Toxin binding to the cell surface occurs at 4°C, but endocytosis of the bound toxin is blocked. Thus, our assay specifically monitored host-toxin interactions at the cell surface. A preliminary control experiment ensured that the extracts did not directly quench the fluorescent signal from FITC-CTB (data not shown). Any reduction in the fluorescent signal obtained from extract-treated cells would therefore reflect an extract-induced inhibition of toxin binding to the cell surface. As shown in Figure 3A, both extracts blocked FITC-CTB binding to the cell surface when co-incubated with the toxin. Pre-treatment of the cell surface with grape extract, followed by FITC-CTB incubation in the absence of extract, did not substantially inhibit toxin binding (Fig. 3B). This indicated that the extracts bound directly to the toxin rather than to the host cell. Surprisingly, the extracts could even strip pre-bound toxin from the plasma membrane: a greatly attenuated FITC-CTB signal was obtained when cells were exposed to FITC-CTB for 30 min at 4°C before the addition of grape extract for another 30 min at 4°C (Fig. 3C). In our final variation on the toxin binding assay, extract and FITC-CTB were mixed in the absence of cells. The mixture was subjected to overnight dialysis with a 3500 MWCO filter, and the retained FITC-CTB was then applied to cultured cells. This procedure recorded a substantial inhibition of toxin binding for the grape seed extract but not for the grape pomace extract (Fig. 3D). Thus, at least one compound in the seed extract exhibited a high affinity interaction with FITC-CTB that allowed it to be retained after overnight dialysis. In contrast, anti-toxin agents in the pomace extract exhibited lower affinity interactions with FITC-CTB and were consequently removed by dialysis. Collectively, these results demonstrated that an inhibition of toxin binding to the cell surface was one mechanism involved with extract-induced toxin resistance.

### Grape Extracts Inhibit Intoxication after Toxin Endocytosis

**Figure 4:**
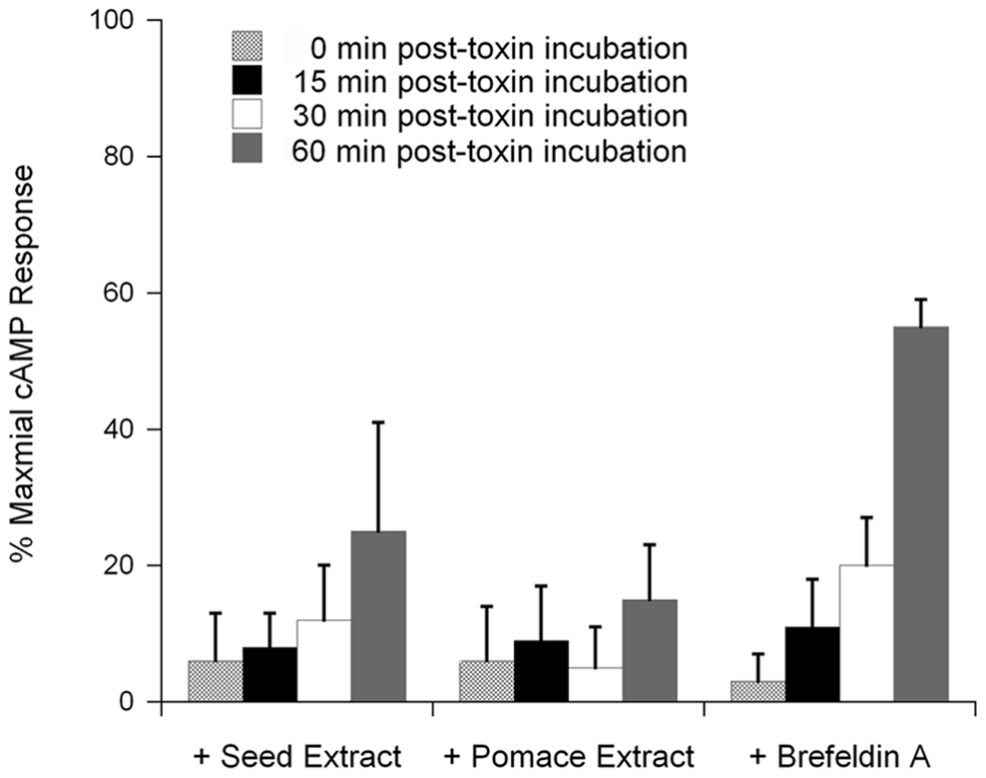
Grape extracts confer protection against CT after endocytosis of the toxin. CHO cells were incubated with CT for 30 min at 4°C. Unbound toxin was removed, and the cells were warmed to 37°C. Grape seed extract, grape pomace extract, or BfA was added to the cells at the time of warming to 37°C (0 min post-toxin incubation) and 15, 30, or 60 min after warming to 37°C. cAMP levels were determined 2 h after the initial warming to 37°C. The maximal cAMP response was obtained from toxin-treated cells incubated in the absence of extract or BfA. The averages ± standard deviations of 3 independent experiments with triplicate samples are shown. At all time intervals, the differences between untreated cells and extract- or BfA-treated cells were statistically significant (1-way ANOVA, *p*<0.01).

Additional cell-based experiments demonstrated that the extracts inhibited host-toxin interactions downstream of the toxin binding event as well (Fig. 4). CHO cells were exposed to CT at 4°C and then warmed to 37°C, a temperature that permits endocytosis of the surface-bound toxin. Extracts were only added 15, 30, or 60 minutes after warming to 37°C. Although delayed exposure to the extract would allow toxin binding and endocytosis to occur, we still detected extract-induced blocks of intoxication. In fact, application of the extracts 30 min after toxin exposure was as effective at blocking intoxication as a co-incubation of the toxin and extract throughout the experiment. A strong inhibitory effect on intoxication was also documented when the extracts were applied 60 minutes after the initial toxin exposure. These results demonstrated that the grape extracts can disrupt intoxication even after toxin internalization into the host cell. BfA also inhibited CT activity against cultured cells but was not as effective as the extracts when applied 30 or 60 min after toxin exposure. The continued efficacy of the extracts at later post-exposure time points suggested that the extracts disrupted host-toxin interactions downstream of the BfA-sensitive trafficking event.

### Grape Extracts do not Inhibit Toxin Transport to the ER or Holotoxin Disassembly

**Figure 5:**
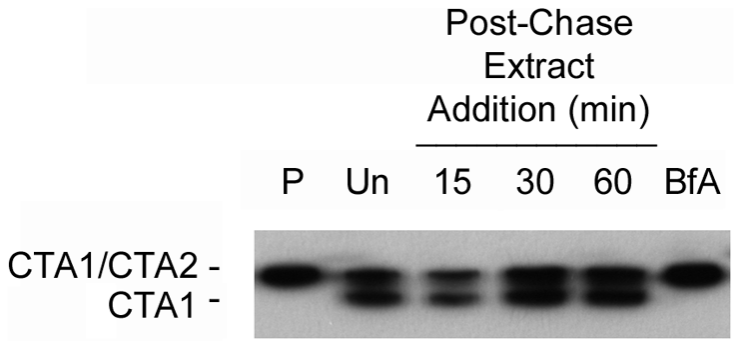
Grape seed extract does not prevent toxin transport to the ER. HeLa cells were incubated with CT for 30 min at 4°C. Unbound toxin was removed, and the cells were warmed to 37°C. Grape seed extract was only added to the cells 15, 30, or 60 min after warming to 37°C. As additional controls, cells were untreated after warming (Un) or were exposed to BfA at the time of warming (BfA). Cell extracts generated after the 4°C pulse labeling (P) or after a total of 2 h at 37°C were separated into membrane and cytosolic fractions. The membrane fractions were resolved by non-reducing SDS-PAGE before Western blot analysis with an anti-CTA1 antibody. Reduction of the CTA1/CTA2 disulfide bond is indicative of toxin transport to the ER.

To determine if the grape extracts blocked CT trafficking to the ER, we monitored the redox status of CTA1. Reduction of the CTA1/CTA2 disulfide bond occurs in the ER [11], [12], so the appearance of reduced CTA1 can be used as an indicator of toxin transport to the ER [15], [18]. HeLa cells were incubated with 1 µg/mL of CT at 4°C for 30 min. Unbound toxin was removed, and the cells were then placed at 37°C to permit endocytosis of the surface-bound toxin. Grape extract was added 15, 30, or 60 minutes after warming to 37°C. With this protocol, the extracts would not block toxin binding to the plasma membrane because they were only added to the medium after endocytosis of the surface-bound toxin had already occurred. CTA1 in the membrane fraction of digitonin-permeabilized cells was collected after a total of 2 hours at 37°C, resolved by non-reducing SDS-PAGE, and visualized with Western blot analysis. As shown in Figure 5, a pool of reduced CTA1 could be detected in the untreated control cells but not in BfA-treated cells. The latter result demonstrated that trafficking to the ER was a prerequisite for reduction of the CTA1/CTA2 disulfide bond. A pool of reduced CTA1 could also be detected in cells treated with grape seed extract at 15, 30, or 60 minutes after warming to 37°C. An identical result was obtained from cells treated with grape pomace extract (data not shown). These observations indicated that the extracts did not inhibit toxin trafficking to the ER, which was somewhat surprising as at least one plant compound has demonstrated inhibitory effects on retrograde toxin transport [45].

**Figure 6:**
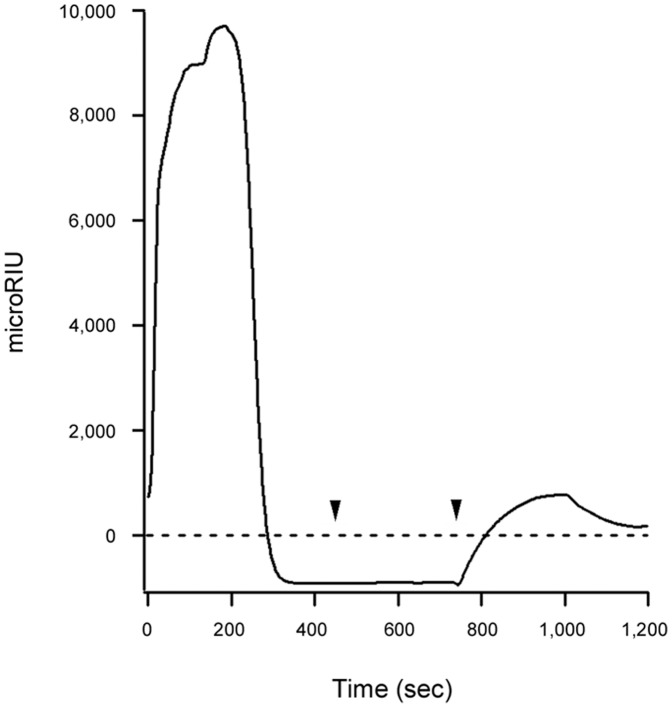
Grape seed extract does not prevent PDI-mediated disassembly of the CT holotoxin. Reduced PDI and grape seed extract were perfused over an SPR sensor coated with the CT holotoxin. The PDI- and extract-containing perfusion buffer was replaced with a buffer containing an anti-CTA1 antibody (first arrowhead), which in turn was replaced with a buffer containing an anti-KDEL antibody (second arrowhead). At the beginning of the experiment, the signal corresponding to the mass of the intact CT holotoxin was set at a baseline RIU value of zero (dotted line).

CTA1 remains associated with the holotoxin through non-covalent interactions after reduction of the CTA1/CTA2 disulfide bond in the ER [46]. Separation of CTA1 from CTA2/CTB_5_, which is a prerequisite for CTA1 translocation to the cytosol, involves the action of PDI [13], [14]. To determine whether the grape extracts disrupt PDI-mediated disassembly of the CT holotoxin, we reconstituted this event on a SPR sensor slide (Fig. 6). The CT holotoxin was appended to a GM1-coated sensor slide, and a baseline measurement which reflected the mass of the intact toxin was recorded. PDI and grape seed extract were then perfused over the CT sensor slide in a reducing buffer. Binding of PDI to the holotoxin increased the mass on the surface of the sensor, and this generated a corresponding increase in the refractive index unit (RIU; the angle of light reflected by the slide). However, the elevated RIU signal quickly dropped below the baseline value corresponding to the mass of the bound holotoxin. The drop in RIU, which occurred despite the continued presence of PDI in the perfusion buffer, indicated that both PDI and a portion of the CT holotoxin had been removed from the plate. We confirmed this interpretation with sequential perfusions of anti-CTA1 and anti-KDEL antibodies over the sensor: a positive signal was obtained with the anti-KDEL antibody (which recognizes the C-terminus of CTA2) but not the anti-CTA1 antibody, demonstrating that PDI had displaced CTA1 from the sensor-bound CTA2/CTB_5_ complex. This phenomenon has been documented by SPR in previous publications as well [13], [16], [18]. Here, we used the assay to demonstrate that grape seed extract does not inhibit PDI-mediated disassembly of the CT holotoxin. Additional experiments confirmed that PDI-mediated holotoxin disassembly could also occur in the presence of grape pomace extract (data not shown).

**Figure 7:**
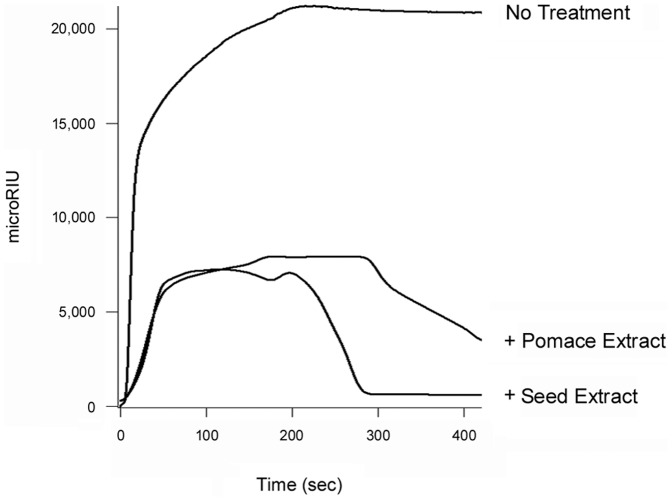
Grape extracts strip CT from lipid bilayers. At time point 0, CT was perfused over a SPR sensor coated with GM1-containing LUVs. Grape seed or grape pomace extract was added to the perfusion buffer at 200 sec in the continued presence of CT. One of two representative experiments is shown.

Grape extracts could strip pre-bound FITC-CTB from the surface of cultured CHO cells (Fig. 3), but they did not strip CT from the GM1-coated SPR sensor slide (Fig. 6). The CT holotoxin reportedly has a higher avidity for GM1 than the CTB pentamer has for GM1 [47], so we performed additional experiments to determine if the differential effects observed in SPR vs. cell culture were due to the use of CT (SPR) vs. FITC-CTB (cell culture). We found that neither grape extract could remove pre-bound CT, CTB pentamer, or FITC-CTB from GM1-coated SPR sensor slides (data not shown). Thus, the differential effects did not arise from use of a CTB pentamer or a FITC-conjugated CTB pentamer. The fluid, complex nature of the plasma membrane (as opposed to an immobilized GM1 monolayer on a SPR sensor) apparently contributes to the ability of grape extract to strip pre-bound toxin from the host cell surface. Different results for in vitro vs. cell-based assays have also been noted for the interaction between ST and its glycosphingolipid Gb3 receptor; these differences have likewise been attributed to the fluidity and complexity of the plasma membrane [48]. To further examine this possibility, we appended GM1-containing LUVs to a SPR sensor slide. The LUVs were formulated to mimic the composition of the lipid raft binding site for CT (see Materials and Methods). Perfusion of CT over the LUV-coated sensor resulted in toxin retention on the slide. Subsequent addition of either grape extract to the perfusion buffer resulted in the displacement of CT from the sensor slide, although the toxin was removed more efficiently by grape seed extract than grape pomace extract (Fig. 7). After removal of either extract from the perfusion buffer, the sensor was able to re-capture a fresh sample of CT that was added to the perfusion buffer (data not shown). This confirmed that the LUVs were retained on the extract-treated sensor. LUVs lacking GM1 were unable to capture CT at the preliminary stage of the experiment, thus demonstrating the specificity of CT binding to GM1-containing membranes (data not shown). Collectively, our data indicated grape extracts can strip GM1-bound CT from fluid lipid bilayers but not from an immobilized GM1 monolayer.

### Grape Extracts Inhibit the Thermal Unfolding of CTA1

**Figure 8:**
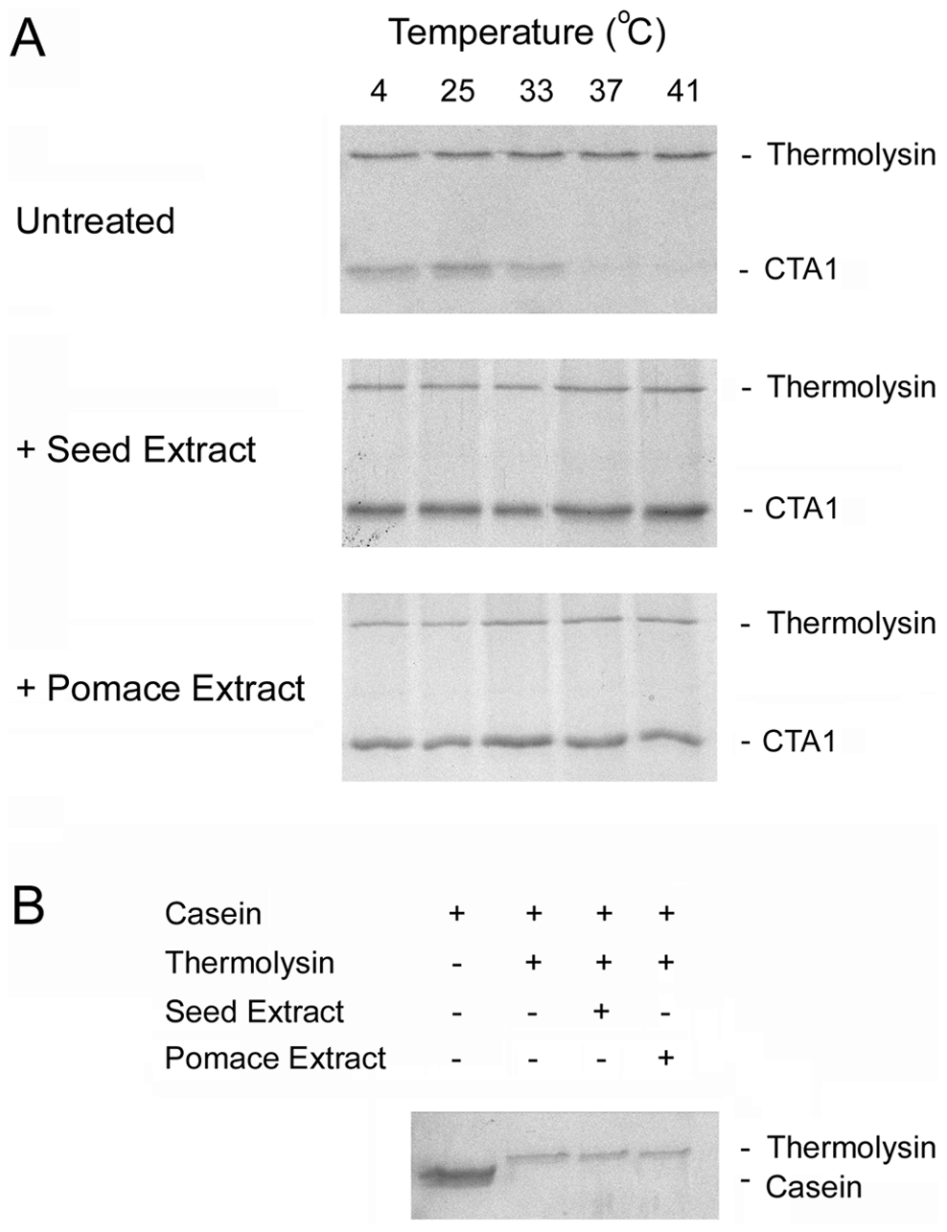
Grape extracts prevent the temperature-induced unfolding of CTA1. (A) A purified CTA1/CTA2 heterodimer was placed in 20 mM sodium phosphate buffer (pH 7.4) containing 10 mM β-mercaptoethanol. Aliquots (1 µg) of the toxin were either left untreated, treated with grape seed extract, or treated with grape pomace extract as indicated. All samples were incubated at the indicated temperatures for 1 h. The toxins were then shifted to 4°C and exposed to the thermolysin protease for 1 h. Samples were resolved by SDS-PAGE and Coomassie staining, which does not visualize the 5 kDa CTA2 subunit. (B) Purified α-casein was placed in 20 mM sodium phosphate buffer (pH 7.4) containing 10 mM β-mercaptoethanol. Aliquots (5 µg) of the protein were incubated from 1 h at 4°C in the absence or presence of thermolysin before visualization by SDS-PAGE and Coomassie staining. Samples exposed to thermolysin were untreated or co-incubated with either grape seed or grape pomace extract as indicated.

An in vitro assay demonstrated that the extracts could alter the structural properties of CTA1. After dissociation from the holotoxin in the ER, the isolated CTA1 subunit unfolds spontaneously at physiological temperature [17]. This unfolding event places CTA1 in a protease-sensitive conformation and triggers its ERAD-mediated export to the cytosol [16], [17]. As shown in Figure 8A, both grape extracts blocked the temperature-induced shift of CTA1 to a protease-sensitive (i.e., unfolded) conformation. Proteolysis of α-casein, a protein with an open and protease-sensitive conformation [49], was not inhibited by the extracts (Fig. 8B). This demonstrated that the extract-induced block of CTA1 degradation was not due to an inhibition of the protease itself. When combined with the results of Figure 3, the protease sensitivity assay demonstrated that both the catalytic and cell-binding components of CT are affected by the extracts. We have previously shown that the thermal stabilization of CTA1 will block toxin export to the cytosol and productive intoxication [15], [16], [18]. Thus, extract-induced resistance to CT may also involve a disruption of toxin translocation to the cytosol.

### Grape Extracts Inhibit CTA1 Translocation to the Cytosol

**Figure 9:**
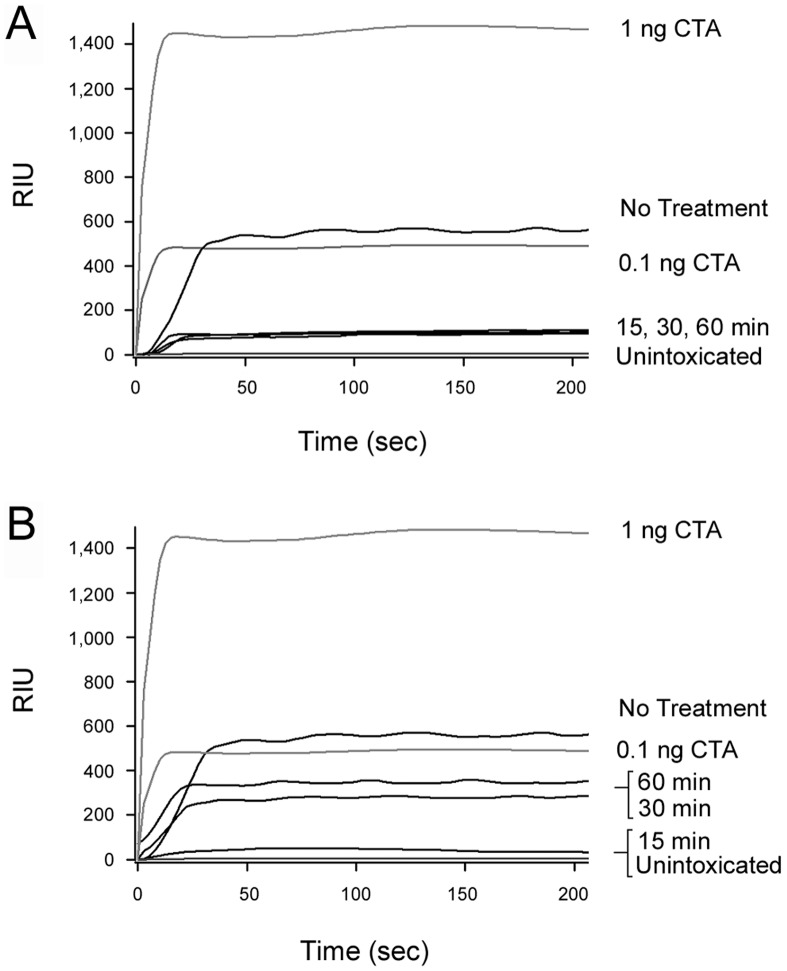
Grape extracts prevent CTA1 translocation to the cytosol. HeLa cells were incubated with CT for 30 min at 4°C. Unbound toxin was removed, and the cells were warmed to 37°C. Grape pomace (A) or grape seed (B) extract was added to the cells 15, 30, or 60 min after warming to 37°C. Cell extracts generated after a total of 2 h at 37°C were separated into membrane and cytosolic fractions, and the cytosolic fractions were perfused over an SPR sensor coated with an anti-CTA1 antibody. The cytosolic fraction from unintoxicated cells was used as a negative control, and CTA standards were used as positive controls. All samples were perfused over the same SPR sensor; the data is presented in two panels for clarity. One of two representative experiments is shown.

To directly examine the potential extract-induced disruption of toxin translocation, we performed the cellular pulse-chase protocol previously utilized to monitor the redox status of cell-associated CTA1. For this assay, the cytosolic fraction of digitonin-permeabilized cells was collected and perfused over an SPR sensor coated with an anti-CTA1 antibody (Fig. 9). Capture of cytosolic CTA1 by the anti-CTA1 antibody would increase the mass on the sensor slide, and this would subsequently be detected as an increase in the RIU. Whereas slightly more than 0.1 ng/mL of CTA1 was detected in the cytosol of untreated cells, only minimal levels of cytosolic toxin were detected from cells treated with grape pomace extract 15, 30, or 60 minutes after the initial toxin exposure (Fig. 9A). The signal generated from cells treated with grape pomace extract was only slightly greater than the baseline signal from the antibody-coated sensor (0 RIU) and the signal obtained from the cytosol of unintoxicated cells. No signal was obtained from the cytosol of cells treated with grape seed extract after 15 minutes of intoxication, although some cytosolic CTA1 was present when grape seed extract was added 30 or 60 minutes after the initial toxin exposure (Fig. 9B). These results correlated well with the toxicity data of Figure 4 which suggested the seed extract was slightly less protective than the pomace extract when applied at 30 or 60 minutes post-intoxication. The data were also consistent with the observed extract-induced inhibition of CTA1 unfolding (Figure 8), which would in turn prevent CTA1 translocation to the cytosol [15], [16], [18].

### Grape Extracts do not Block Anterograde Vesicular Transport of the Dissociated CTA1 Subunit

**Figure 10:**
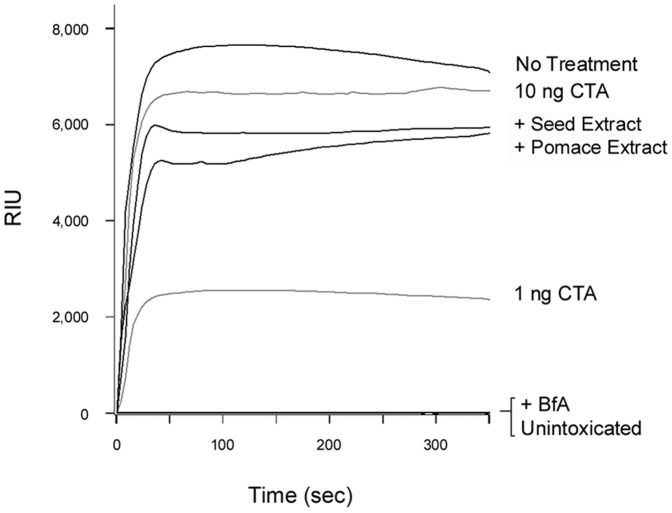
Grape extracts do not prevent the secretion of free CTA1. HeLa cells were incubated with CT for 30 min at 4°C. Unbound toxin was removed, and the cells were warmed to 37°C. BfA was added immediately after warming, and grape pomace or grape seed extract was added to the cells 15 min after warming to 37°C. Media samples collected after a total of 2 h at 37°C were perfused over an SPR sensor coated with an anti-CTA1 antibody. The extracellular medium from unintoxicated cells was used as a negative control, and CTA standards were used as positive controls. One of two representative experiments is shown.

When CTA1 is released from its holotoxin in the ER, a portion of free CTA1 enters the secretory pathway and is released into the extracellular medium [16]. To detect the secreted pool of free CTA1 from untreated and extract-treated cells, we collected media samples from cells used for the translocation assay of Figure 9 as well as from BfA-treated cells. These samples were perfused over an SPR sensor coated with an anti-CTA1 antibody. No signal was obtained from the medium of unintoxicated control cells or from the medium of intoxicated, BfA-treated cells (Fig. 10). Both anterograde and retrograde vesicular transport pathways are disrupted by BfA, which consequently prevents the secretion of free CTA1 [16]. Media samples from CT-treated cells incubated in either the absence or presence of grape extract produced roughly equivalent SPR signals, indicating that a post-toxin exposure to grape extract did not substantially inhibit secretion of the dissociated CTA1 subunit. To ensure the extracellular pool of toxin represented free CTA1 and not holotoxin-associated CTA1, we used an anti-CTB antibody to immunodeplete the CTB pentamer and CT holotoxin from parallel media samples before their perfusion over the CTA1 sensor. This procedure did not result in any loss of signal (data not shown). Such a result demonstrated that, consistent with previous observations [16], the extracellular pool of toxin was free CTA1 and not CT holotoxin. Thus, the grape extracts did not block (i) retrograde CT transport to the ER (see also Fig. 5); (ii) CTA1 dissociation from CTA2/CTB_5_ (see also Fig. 6); or (iii) anterograde secretory transport of free CTA1 from the ER to the extracellular medium.

### Grape Extracts Inhibit the Enzymatic Activity of CTA1

**Figure 11:**
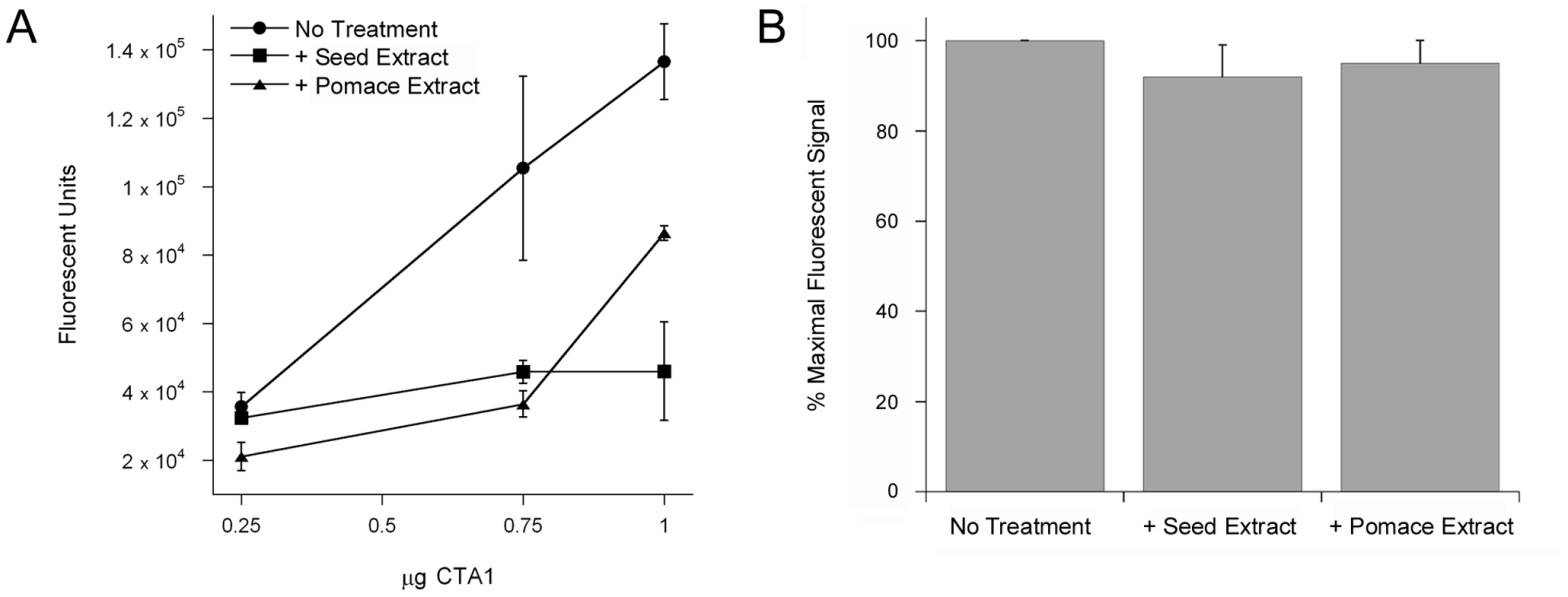
Grape extracts inhibit the ADP-ribosyltransferase activity of CTA1. (A) Dilutions of CTA1 mixed with DEA-BAG in the presence or absence of grape extract were placed at 25°C for 2 h. The ADP-ribosylation of DEA-BAG was then assessed by fluorometry; increasing fluorescent units correspond to increasing levels of substrate modification. Data are presented as the averages ± ranges of two replicate samples per condition. One of two representative experiments is shown. (B) DEA-BAG was placed in buffer lacking or containing either grape seed or grape pomace extract. The intrinsic fluorescence of DEA-BAG and extract-treated DEA-BAG was then assessed by fluorometry. Data are presented as percentages of the value obtained from untreated DEA-BAG, which was used at a concentration that did not saturate signal detection. The averages ± standard deviations of 3 independent experiments with triplicate samples are shown.

Plant extracts and phenolic compounds have predicted [50] or demonstrated [32], [33], [45] inhibitory effects on the ADP-ribosyltransferase activity of CTA1. An in vitro assay was accordingly used to monitor the enzymatic function of CTA1 in the presence of grape seed or grape pomace extract. As shown in Figure 11A, both extracts affected CTA1 activity: the dose-dependent modification of DEA-BAG, a synthetic substrate for the ADP-ribosylation activity of CTA1, was inhibited but not completely blocked in the presence of either grape extract. Control experiments demonstrated that neither grape extract directly quenched the intrinsic fluorescence of the DEA-BAG substrate which was used to monitor toxin activity (Fig. 11B, see also Materials and Methods).

## Discussion

The grape seed and grape pomace extracts used in this study are sold as nutritional supplements under the names MegaNatural Gold and MegaNatural GSKE, respectfully. Both extracts are Generally Recognized as Safe by the United States Food and Drug Administration. Studies with human volunteers have shown that the extracts retain bioactivity after oral ingestion of up to 600 mg/day of extract [51], [52]. In this study, we demonstrated the potential utility of grape extract as a therapeutic to prevent and possibly treat cholera.

Like other plant extracts [31], [43], the grape seed and grape pomace extracts blocked CT binding to the cell surface. This effect apparently involved an interaction with the toxin rather than the host, as pretreatment of the host cell with grape extract did not prevent the subsequent binding of FITC-CTB in the absence of extract. Furthermore, at least one compound in the grape seed extract exhibited a high affinity interaction with CTB that allowed it to be retained with the toxin (and to inhibit toxin binding) after an overnight dialysis. A post-dialysis block of FITC-CTB binding to the target cell was not observed when the toxin was treated with grape pomace extract, thus demonstrating that the two grape extracts contain different anti-toxin components or different effective concentrations of the same anti-toxin component.

We also noted that both extracts could strip pre-bound toxin from the plasma membrane. To the best of our knowledge, this is the first demonstration of CT removal from the cell surface by a non-toxic plant extract. The mechanism by which CTB is removed from the cell surface remains to be determined and is of high interest, but the responsible compound(s) will need to be isolated before the molecular details of this event can be established. Still, the observations reported here suggest grape extracts could provide a post-exposure medicinal benefit by stripping bound CT from the surface of target enterocytes.

Our intoxication assays also indicated the extracts could function as effective inhibitors when applied after toxin exposure. Extract-treated cells were highly resistant to CT, even when the extracts were applied an hour after toxin challenge. This demonstrated that the extracts can disrupt CT intoxication downstream of toxin binding and endocytosis. Our work identified a number of these downstream events. In vitro, both extracts blocked the temperature-induced shift of CTA1 to a disordered, protease-sensitive conformation. Previous work has shown that thermal disordering of the dissociated CTA1 polypeptide is a prerequisite for its translocation to the cytosol, and our in vivo translocation assay indeed detected an inhibition of CTA1 translocation in extract-treated cells. The ADP-ribosyltransferase activity of CTA1 was also inhibited by the grape extracts.

Our collective data thus demonstrated that multiple steps of the CT intoxication process are inhibited by grape extract. Events involving both the A1 subunit and the B pentamer were affected. This may explain, in part, the potent anti-toxin properties of the extracts: productive intoxication of extract-treated cells would require CT to overcome disruptions of (i) CTB binding to the cell surface; (ii) unfolding and translocation of the ER-localized CTA1 subunit; and (iii) CTA1 enzymatic activity. Each of these events involves CT rather than the host cell, which is consistent with the non-toxic nature of the extracts [30] and the lack of extract-induced alterations to adenylate cyclase signaling, redox status, protease function, and anterograde/retrograde vesicle trafficking.

The grape extracts affected many, but not all, steps of the CT intoxication process. Retrograde transport of the toxin to the ER was not blocked in extract-treated cells. Likewise, PDI could still dislodge CTA1 from CTA2/CTB_5_ in the presence of grape extract. A portion of the dissociated CTA1 subunit moves from the ER to the Golgi apparatus and is released into the medium; this event was not substantially affected by the grape extracts. Previous studies have suggested the polyphenolic constituents of plant extracts can induce non-specific protein aggregation that may be at least partially responsible for toxin resistance [31], [45], [53]. However, grape extracts appear to generate specific inhibitory effects because they only disrupt a subset of intoxication events. It is possible that the concentrations of individual polyphenolic compounds in our grape extracts (used at 100 µg/mL concentrations for all experiments) were too low to induce non-specific protein aggregation. For example, peptides are only precipitated by the plant polyphenol epigallocatechin gallate when it is present at concentrations greater than ∼4.5 mg/mL [53]. The tannin-induced precipitation of Staphylococcal α-toxin likewise required a polyphenol concentration of 400 µg/mL [54], while the partial precipitation of CT by resveratrol required a polyphenol concentration of 46 µg/mL [45]. The lack of grape extract-induced protein precipitation is also consistent with the fact that our extracts are not toxic when applied to cells at concentrations up to 500 µg/mL [30].

Grape seed and grape pomace extracts have known chemical compositions: they are highly enriched in polyphenolic compounds which exhibit medicinal properties for heart disease and other disorders [51], [52], [55], [56]. Polyphenolic compounds from other plant extracts have demonstrated anti-toxin activities against CT, *Helicobacter pylori* VacA, Staphylococcal α-toxin, and anthrax lethal factor [31]–[33], [42], [44], [45], [54], [57], [58]. Furthermore, computational analysis has predicted an inhibition of CTA1 catalytic activity by the catechin family of polyphenolic compounds [50]. These observations strongly suggest a polyphenolic compound(s) in the grape extract is responsible for the multiple disruptions of host-toxin interactions that result in the inhibition of CT/LT activity against cultured cells and intestinal loops. In support of this hypothesis, we found that cells treated with a defined cocktail of purified phenolic compounds exhibited substantial resistance to CT (Fig. 1C). Future screens of individual polyphenolic compounds and cocktails of defined polyphenolic composition will identify the specific anti-toxin components of the grape extracts. The post-dialysis retention of an anti-toxin compound(s) with the CTB pentamer (Fig. 3D) should also facilitate inhibitor identification.

In summary, two over-the-counter nutritional supplements strongly inhibit CT/LT activity against cultured cells and intestinal loops. Both of these grape extracts target a subset of toxin-specific events that are required for productive intoxication. In vivo intoxication is likely an asynchronous process, with individual enterocytes exhibiting cytopathic effects at different stages of infection. In theory, grape extracts could therefore represent a new anti-toxin therapeutic with medicinal value as a prophylactic or even after pathogen/toxin exposure.
